# The miniature genome of broad mite, *Polyphagotarsonemus latus* (Tarsonemidae: Acari)

**DOI:** 10.1038/s41597-024-03579-4

**Published:** 2024-07-09

**Authors:** Muthugounder Mohan, Neenu Augustine, Selva Babu Selvamani, Aneesha P. J., Upasna Selvapandian, Jyoti Pathak, Gandhi Gracy R., Venkatesan Thiruvengadam, Sushil S. N.

**Affiliations:** 1 Division of Genomic Resources, ICAR- National Bureau of Agricultural Insect Resources, Hebbal, Bengaluru 560024 India; 2 Present Address: School of Agricultural Innovations and Advanced Learning (VAIAL), Vellore Institute of Technology, Tamil Nadu, 632014 India

**Keywords:** Evolutionary developmental biology, Entomology

## Abstract

The broad mite, *Polyphagotarsonemus latus* (Tarsonemidae: Acari) is a highly polyphagous species that damage plant species spread across 57 different families. This pest has developed high levels of resistance to some commonly used acaricides. In the present investigation, we deciphered the genome information of *P. latus* by PacBio HiFi sequencing. *P. latus* is the third smallest arthropod genome sequenced so far with a size of 49.1 Mb. The entire genome was assembled into two contigs. A set of 9,286 protein-coding genes were annotated. Its compact genome size could be credited with multiple features such as very low repeat content (5.1%) due to the lack of proliferation of transposable elements, high gene density (189.1/Mb), more intronless genes (20.3%) and low microsatellite density (0.63%).

## Background & Summary

The chelicerate mites and ticks, belonging to the Acari group, are the second most diverse group of animals on the earth after insects. The spiders, mites, ticks, and scorpions together constitute one of the mega-diverse arthropod lineages with global scale distribution and significant economic and ecological importance. The class Acari comprises two superorders namely Acariformes and Parasitiformes. Acari diverged from other arthropod lineages approximately 400 million years ago and formed a separate lineage^1^. Under Acariformes, the order Trombidiformes represents major phytophagous superfamilies of mites such as Tetranychoidea and Tarsonemoidea^2^. Eriophyoidea, the yet another superfamily with phytophagous mites is placed under another order called Sarcoptiformes as per the recent classification^3^. Mites comprise many notorious pests of agricultural and veterinary importance. They can thrive across a wide range of habitats and display great diversity in their evolution.

**Fig. 1:**
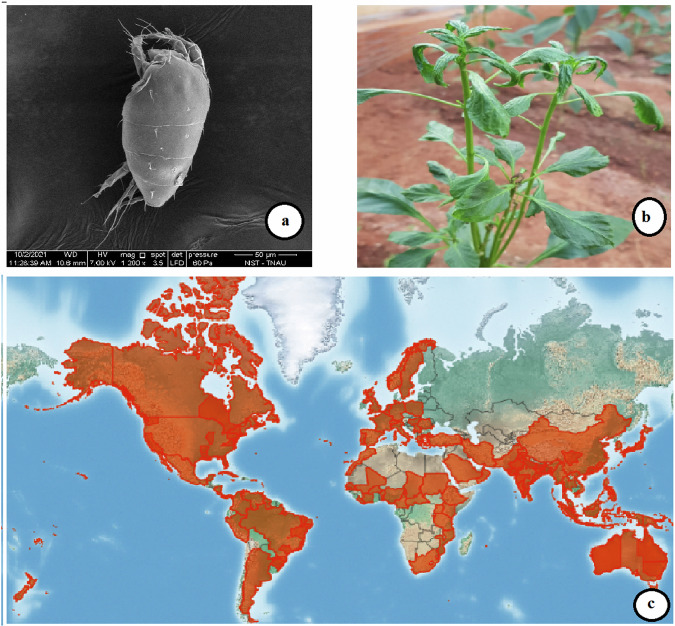
(**a**) Scanning Electron Microscopy (SEM) image of broad mite female, *Polyphagotarsonemus latus*(1200x magnification), (**b**) Damage by broad mite on hot pepper and (**c**) Distribution map - Courtesy: Centre for Agriculture and Biosciences International (CABI, UK).

Phytophagous mites classified under the family Tarsonemidae contain about 40 genera and more than 500 described species, of which the broad mite or yellow mite, *Polyphagotarsonemus latus* (Banks) (Fig. 1a) is a serious pest of more than 250 crop plants of commercial importance that spread across 57 plant families^4^. The crops such as hot (Fig. 1b) and sweet peppers, mulberry, citrus, cotton, tea, mango, jute, and potato are severely damaged among others. At present, it has become a well-established pest across all six zoogeographical regions worldwide, namely, Australia, Asia, Africa, North America, South America, and the Pacific Islands^5^ (Fig. 1c). Like many other mites, *P. latus* reproduces through the haplo-diploidy system with a female-biased sex ratio^4^. The males are haploid (n = 2) and produced by arrhenotokous parthenogenesis and the females are diploid (2n = 4) and produced by fertilized eggs^6^. It is equally prevalent in tropical, subtropical, and greenhouse environments owing to several intrinsic and extrinsic factors^7–9^. Due to its microscopic nature, its occurrence is evident only after substantial injury is caused to the plants. Gregory and Young^10^ emphasized a positive relationship between genome size and body size in mites, while also noting that certain acarine genomes exhibit remarkably low levels of DNA content. *P. latus* exhibits a significantly smaller size (<0.2 mm), amounting to approximately half or even less than that of other mites such as *Tetranychus urticae*. The minute body size of *P. latus* coupled with many other genomic features aligns well with this observation.

Chemical control with synthetic acaricides remains the common management strategy adopted by crop growers. The intensive and widespread use of acaricides coupled with shorter life cycles and unusual modes of reproduction has favoured many mite and tick species around the world to evolve resistance against acaricides with different modes of action^11–13^. In India, more than two dozen acaricides under 12 different modes of action were officially used for the management of phytophagous mites. Due to their indiscriminate/overuse, the occurrence of high levels of acaricide resistance in field-collected *P. latus* populations was documented^14,15^.

To date, genomes of 39 mites and 17 ticks have been sequenced (NCBI, accessed 27 November 2023) including seven species of phytophagous mites namely, *T. urticae*^16^, *T. cinnabarinus*, *T. truncatus*^17^ (Spider mites: Tetranychoidea: Tetranychidae), *Panonychus citri*^18^ (Citrus red mite: Tetranychoidea: Tetranychidae), *Aculops lycopersici*^19^ (Tomato russet mite: Eriophyoidea: Eriophyidae), *Fragariocoptes setiger*^20^ (Gall mite: Eriophyoidea: Eriophyidae) and *Halotydeus destructor*^21^ (Redlegged earth mite: Eupodoidea: Penthaleidae).The majority of the mite genomes are smaller as compared to ticks. The *de novo* draft genome of *P. latus* brought off in this study is the third smallest arthropod genome sequenced so far and the first deciphered genome under the Acari family Tarsonemidae. Its genome has been assembled into just two contigs, the lowest among the mite species. The genome has very low repeat content (5.1%) due to a lack of proliferation of transposable elements. Although the genome codes only 9,286 protein-coding genes, it has nothing to do with the pest’s adaptability to xenobiotics^14,15^. CAFE analysis revealed that *P. latus* exhibits one of the highest rates of gene family contractions and a lower rate of gene family expansions. The assembled genome of *P. latus* holds many novel features both froma phylogenetic point of view as well as developing new acaricidal molecules and novel pest management strategies.

## Methods

### Establishment and maintenance of *P. latus* colony

*P. latus* was originally collected from hot pepper plants (Fig. 1b) cultivated near Bengaluru, India (12.6254°N, 77.2319°E) during July 2020, and subsequently an iso-female colony was established (NBAIR-GR-TAR-01a) under laboratory conditions. This colony was maintained on potted mulberry plants (*Morus alba*; variety: V1) in a growth chamber since then at ICAR-National Bureau of Agricultural Insect Resources, Bengaluru, India. The identity was further confirmed by DNA barcoding. A 631 bp long *COI* sequence was deposited in the NCBI-GenBank (ON103156). The amplified *COI* sequence and the specimen details were submitted to the BOLD database V4 (BIN No. AED8321).

### DNA isolation, library preparation, and sequencing

More than 5,000 adult females from the laboratory-reared susceptible iso-female colony were individually hand-picked from the infested leaves under the microscope. The collected mites were starved for a brief time and then frozen in liquid nitrogen. High-quality, high molecular-weight DNA was extracted using the CTAB method^22^. The extracted DNA was dissolved in the TE buffer and sent to Nucleome Informatics Pvt. Ltd. (Hyderabad, India) for library preparation and sequencing. DNA was sheared to 15-20 Kb with the g-tube system (Covaris). SMRTbell^®^ gDNA Sample Amplification Kit was used to amplify the DNA. Approximately 600 ng of amplified DNA was used to generate a HiFi SMRT bell library for one Sequel II SMRT Cell using SMRTbell Express template preparation kit 2 (PacBio, USA). Size selection (20 kb) was performed using the BluePippin system (Sago Science). In all the steps, DNA was quantified by Qubit Fluorometer (ThermoFisher Scientific) and quality was checked by Femto Pulse (Agilent Technologies). The libraries were sequenced on the PacBio Sequel II platform. The subreads were used to call the CCS reads using the SMRT link v10.2 (PacBio, USA) to produce HiFi reads by CCS software (https://github.com/PacificBiosciences/ccs). The following settings were used: minimum number of passes: 1, minimum accuracy 0.9 with quality Q20 and above. From the two runs, 40.9 Gb and 38.8 Gb of raw data were generated with N50 values of 8,239 and 6,063, respectively (Table 1). A total of 186,760 HiFi reads were generated with an average read length of 11.6 Kb (Table 1).

**Table 1:** Complete PacBio sequencing metrics of the *P. latus* genome.

Statistics	Run1	Run2
Polymerase Reads	645,048	1,701,502
Polymerase Read Bases	40,916,450,523	38,831,028,329
Polymerase Read Length (mean) (bp)	63,431	22,821
Polymerase Read N50 (bp)	166,855	97,481
Subread Length (mean) (bp)	7,997	5,341
Mean of Longest Subread Length (bp)	19,803	9,262
Subread N50 (bp)	8,239	6,063
Longest Subread N50 (bp)	49,484	24,937
Unique Molecular Yield (bp)	11,430,072,320	14,905,624,576
Total HiFi Reads	186,760	
Median HiFi Read Length (Kb)	11.6	
Q20%	98.94	
Q30%	97.82	
Coverage	28x	

### Contamination removal, contig level assembly, and assembly polishing

The microbial contaminations were filtered out using Kraken2 v2.1.2^23^ and adapters were removed through a similarity search against the NCBI UniVec database using Blastn. The reads were further mapped against the host (mulberry) genome (GenBank assembly accession no. GCA_012066045.3) using Minimap2 v2.24^24^ to eliminate the host plant DNA contamination that resulted in the removal of 1,494 reads.The filtered HiFi reads were assembled into contigs using the default parameters of Hifiasm v0.16^25^, an efficient and fast haplotype-resolved*de novo* assembler especially for PacBio HiFi reads. From the resulting partially phased genome, we took hap2 for the downstream analysis as the organism is homozygous (from the k-mer plot generated by the assembler) and the number of contigs is less compared to the hap1 assembly. To improve the contig assembly, HiFi reads were aligned back to the hap2 genome assembly using pbmm2 v1.5.0, and an aligned sorted bam file was generated, which was used to polish and assemble the hap2 assembly using gcpp v1.9.0. The phased genome has beenassembled into two contigs with an assembly size of 49.1 Mb and N50 value of 30.90 Mb (Table 2).

**Table 2:** Contig level assembly statistics of *P. latus* genome.

Parameter	Statistics
Contigs	2
Largest contig	30,903,301
Total length (bp)	49,135,586
GC (%)	20.01
Contig 1	30,903,301
Contig 2	18,232,285
L50	1
L75	2
# Ns per 100 Kb	0

### Estimation of genome size and completeness

The k-mer frequency distribution histogram was constructed using a jellyfish v 2.3.0 program^26^. Histogram along with read length and k-mer length were used as inputs in the program Genomescope v 2.0 (k = 21) for the estimation of genome size, level of repeats, and heterozygosity. The assembly length was observed to be similar to the length estimated by k-mer analysis (49.3 Mb) with the lowest error rate of 0.27 percent. The average heterozygosity rate was estimated at 0.16 percent (Table S1; Fig. S1).

### Repeat identification and genome masking

For the identification and accurate compilation of sequence models representing all of the unique transposable element (TE) families dispersed in the assembled genome, RepeatModeler2^27^ v2.0.4 was used which is an automated pipeline employing different algorithms for the repeat identification. After repeat identification, 2,497,763 bp were masked in the assembled genome. The dominant repeat elements were simple repeats accounting for 2.49%, followed by low complexity repeats (1.62%), total interspersed repeats (0.97%), retroelements (0.27%), and LINES (0.24%) (Table 3).

**Table 3:** Repeat element statistics of *P. latus* genome.

Repeat element	No. of elements	Length occupied	Percentage of sequence
Retro elements	303	132,143 bp	0.27%
LINEs	283	116,540 bp	0.24%
L2/CR1/Rex	19	10,107 bp	0.02%
R1/LOA/Jockey	253	103,975 bp	0.21%
LTR elements (Ty1/Copia)	20	15,603 bp	0.03%
Total interspersed repeats	—	477,200 bp	0.97%
Simple repeats	27,168	1,222,197 bp	2.49%
Low complexity	14,910	794,336 bp	1.62%
RNA repeats	26	4,030 bp	0.01%
Unclassified	1,115	345,057 bp	0.70%

### Gene prediction

The genic and intergenic regions in the assembled genome were predicted using Genemark-ES v2^28^ and Augustus v3.4^29^. Genemark-ES uses the heuristic method of initialization of the hidden semi-Markov model algorithm for finding the maximum likelihood parse of sequence into coding and non-coding regions and also does iterative self-training on sequences, whereas Augustus was trained by pre-trained species from the BUSCO^30^ analysis from the lineage arthropoda_odb10. Also, RNA-Seq of iso-female colonies of acaricide-resistant and susceptible populations were used as evidence for the prediction of genemodels. Approximately 9,286 and 7,787 genes were predicted from the genome assembly using Genemark-ES and Augustus, respectively (Table S2). The genes predicted from the Genemark-ES were used for the annotation. A total of 22,909 introns were predicted in the entire genome with 20.3% of the genes being intronless. A total of 32,195 exons were detected and the number of exons per gene varied from 1–33 (Table S3).

### Functional Annotation and Gene-Ontology

The predicted protein sequences were further annotated with the eggNOG^31^, nr^32^, KEGG^33^, and InterPro^34^ databases. The corresponding Gene Ontology terms (GO) of identified gene sequences were predicted using the eggNOG mapper v2.1.9^35^. A total of 1,274 genes were annotated by BLAST + v2.13.0^36^ againstthe NCBI nr database; 6,964 genes were annotated by Interproscan v5.60.92 and homology search against the KEGG database assigned 4,984 genes with their corresponding pathways. Gene ontology annotation classified 5,420 genes into three GO classes namely biological process, molecular function, and cellular component.

### Prediction of RNA species and microsatellites

To find the different RNAs present in the genome, the Infernal^37^ (INFERence of RNA ALignment)tool v1.1.4 was used with the default parameters. We used cmscan locally to search the Rfam CM libraries against the *P. latus* genome. The RNA species classification revealed a tRNA count of 102 and a miRNA count of four. Further, 5S_rRNA, 5.8S_rRNA, small subunit ribosomal RNA (SSU_rRNA_eukarya), and large subunit ribosomal RNA (LSU_rRNA_eukarya) were observed to be 25, 17, 16, and 16 in numbers, respectively (Table S4). To identify the microsatellite repeats in the assembled genome, Krait v1.4.0^38^, a robust and ultrafast tool was employed. The minimum repeats for each perfect Simple Sequence Repeats (SSR) type were set to 12 for mono-, 7 for di-, 5 for tri, 4 for tetra-, 4 for penta-, 4 for hexa- and motif standardization level was set to Level 3. The total number of perfect SSRs was 23,116 which accounted for 0.64% of the genome (Table S5). Among the perfect microsatellites, tri-nucleotide microsatellites were the most abundant (324), followed by tetra-nucleotides (122), di-nucleotides (73), penta-nucleotides (38) and hexa-nucleotides (26). The most abundant SSR motifs were AAG (37.18%), followed by AAAT (30.53%), ATC (29.49%), and AAT (17.03%) (Table S6).

### Orthologous gene family identification, and CAFÉ analysis

For the single copy orthologous gene identification, genomic information of eight species namely, broad mite (*Polyphagotarsonemus latus*) predatory mite (*Metaseiulus occidentalis*); two-spotted spider mite (*Tetranychus urticae*); tomato mite (*Aculops lycopersici*); common house spider (*Parasteatoda tepidariorum*); social velvet spider (S*tegodyphus mimosarum*) and black-legged tick (*Ixodes scapularis*) were used as ingroup and fruit fly (*Drosophila melanogaster*) was used as outgroup reference. The proteomes of these organisms were collected from NCBI (ftp://ftp.ncbi.nlm.nih.gov/) and Ensembl genome repositories (ftp://ftp.ensemblgenomes.org/).

OrthoVenn v3^39^ is a comprehensive platform for comparative genomics, designed to identify orthogroups and orthologs. Blastp was used for sequence homology search; MUSCLE v5.1^40^ and FastTree2-v2.1.11^41^ were used for multiple sequence alignment; and tree inference for the phylogeny construction, respectively. The OrthoVenn v3 generated 14,646 orthologous clusters with 225 overlaps and 669 single-copy clusters (Fig. S2, S3). A total of 145,984 proteins (proteome of *P. latus* and references) were present of which 27,969 (19.16%) were singletons. CAFE^42^ v5.0 and OrthoMCL v2^43^ algorithm were used for grouping orthologous protein sequences with a P-value threshold of 0.05 to analyse the gene family expansions and contractions in *P. latus* and its related species. The expansion and contraction of gene family analysis revealed that among the eight arthropods analysed, *P. latus* exhibits one of the highest rates of gene family contractions (211) and a lower rate of gene family expansions (43) (Fig. S4).

The GO enrichment analysis using OrthoVenn v3 with P-value of 0.05 of significantly contracted gene families in *P. latus* included xenobiotic metabolic process (GO:0006805), xenobiotic transport (GO:0042908), glucosylceramide catabolic process (GO:0006680), ubiquitin-dependent protein catabolic process (GO:0006511), retinol metabolic process (GO: 0042572), wing disc development (GO: 0035220), visual learning (GO: 0008542) and segmentation (GO: 0035282). The GO terms related to biological processes like cholesterol metabolic process (GO:0008203), lipid catabolic process (GO:0016042), and neuron projection morphogenesis (GO:0048812) were also contracted.

The GO enrichment of the significantly expanded gene families included those responsible for xenobiotic detoxification enzymes like glutathione transferase activity (GO:0004364), UDP-glycosyltransferase activity (GO:0008194), serine-type carboxypeptidase activity (GO:0004185), glucuronosyltransferase activity (GO:0015020), and metalloexopeptidase activity (GO:0008235). The GO terms related to biological processes like testicular fusome organization (GO:0030724), antibiotic metabolic process (GO:0016999), and regulation of store-operated calcium entry (GO:2001256) were also expanded.

## Data Records

The PacBio raw data, genome assembly, and annotation files have been submitted to NCBI under the bioproject ID: PRJNA904956; the assembly accession no: GCA_040055235.1^44^ and SRA ID:SRX19915179,SRX19738152^45^. The transcriptome reads used for validation of genome accuracy have been submitted to NCBI SRR21762741 to SRR21762743 by our previous study^46^. The genome assembly accession IDs of species used in comparative studies were fruit fly (*Drosophila melanogaster*); predatory mite (*Metaseiulus occidentalis*) (GCA_000255335.2); two-spotted spider mite (*Tetranychus urticae*)(GCA_000239435.1); tomato mite (*Aculops lycopersici*) (Eriophyoidea: Eriophyidae)(GCA_015350385.1); common house spider (*Parasteatoda tepidariorum*) (GCA_000365465.3); spider (S*tegodyphus mimosarum*) (GCA_000611955.2); black-legged tick(*Ixodes scapularis*) (GCF_016920785.2); carmine spider mite(*Tetranychus cinnabarinus)*(GCA_022266195.1); spider mites (*Tetranychus truncatus)* (GCA_028476895.1); American house dust mite (*Dermatophagoides farinae*) (GCA_020809275.1); European house dust mite (*Dermatophagoides pteronyssinus*) (GCA_001901225.2); red-legged earth mite (*Halotydeus destructor*) (GCA_022750525.1) and human (*Homo sapiens*) (GCA_000001405.29). The gene annotation, gtf, and sequences file of *P. latus* are shared in figshare 10.6084/m9.figshare.25825984.v1^47^.

## Technical Validation

Completeness and accuracy of the assembled genome were assessed using Benchmarking Universal Single-Copy Orthologs (BUSCO v5)^30^ analysis using the lineage eukaryota_odb10, arthropoda_odb10, and arachnidae_obd10 datasets as orthologs reference, n = 255, 1,013 and 2,934 respectively and by mapping of RNA-Seq reads against the assembled genome. Estimates of the completeness of the draft genome assembly by BUSCO assessment located 92.9%, 86.9% and 83.4% of the eukaryotes single-copy orthologs (92.5% complete single copy and 0.4% duplicated), arthropod single-copy orthologs (84.3% complete single copy and 0.9% duplicated) and arachnid single-copy orthologs searched (82.0% complete single copy and 1.72% duplicated), respectively (Table S7; Fig. S5). The mapping of RNA-Seq reads against the genome was performed using the STAR^48^ aligner v2.7.10b with the default parameters. The mapping percentage of 96.44 was observed for the mapping of RNA-Seq reads against the assembled genome. The statistics indicate that *P. latus* genome assembly is comparable to any other chelicerate genomes concerning the number of assembled contigs and completeness.

### Supplementary information

Table S1, Table S2, Table S3, Table S4, Table S5, Table S6, Table S7

Fig. S1, Fig. S2, Fig. S3, Fig. S4, Fig. S5

## Data Availability

In the current study, no custom scripts were used. All the data processing was done using the guidelines standard pipelines and bioinformatics tools given in the Methods section.
