# Supplementary material for: The miniature genome of broad mite, Polyphagotarsonemus latus (Tarsonemidae: Acari)
Source: Sci Data. 2024 Jul 9;11:748. doi: 10.1038/s41597-024-03579-4 (PMC11233664; doi:10.1038/s41597-024-03579-4)
Supplement: Supplementary file 1 — Table S1, Table S2, Table S3, Table S4, Table S5, Table S6, Table S7 [file 41597_2024_3579_MOESM1_ESM.pdf]

Table S1. Genome properties *of P. latus* using K-mer (K-21) analysis through GenomeScope from PacBio long read

Genome statistics	minimum	maximum
Heterozygosity (%)	0.0153812	0.0173328
Genome Haploid Length (bp)	49,327,226	49,339,730
Genome Repeat Length (bp)	4,657,623	4,658,804
Genome Unique Length (bp)	44,669,603	44,680,926
Model Fit (%)	98.1473	99.0838
Read Error Rate (%)	0.269971	0.269971

Table S2. Gene prediction statistics of *P. latus* genome by Augustus and Genemark-ES

**Annotation**	**Augustus**	**GeneMark-ES**
Introns	23,219	22,909
Genes	7,787	9,286
Transcripts	7,787	9,286
CDS	31,006	32,195

Table S3. Comparison of genome metrics of mite species, with *D. melanogaster,* and *H. sapiens*

Species	Reference	Genome size (Mb)	Protein coding genes	% intronless	Gene density	Coding %
P. latus	This study	49.1	9,286	20.3	189.1	31.6
A. lycopersici	Greenhalgh et al. (2020)	32.5	10,263	83.67	315.4	42.26
D. pteronyssinus		70.7	12,530	25.29	177.1	35.26
T. urticae		90.8	19,086	18.26	209.6	22.1
M. occidentalis		151.9	17,310	24.97	113.9	15.25
D. melanogaster		143.7	13,931	16.37	96.9	15.6
H. sapiens		3,088.3	19,636	6.74	6.4	1.1

% intronless: Percentage of coding genes with no introns predicted using Genomictools; Gene density: The ratio of number of protein coding genes per million base pairs (Mb); Coding %: Percentage of genomes in coding regions by GAG tools (Genome annotation Generator); Genome size estimation was based on k-mer analysis and protein coding genes were predicted using maker software.

Table S4. RNA classification statistics of genome of *P. latus* using Infernal pipeline

**Classification**	**Count**
tRNA	102
miRNA	4
LSU_rRNA_eukarya (large subunit ribosomal RNA)	16
SSU_rRNA_eukarya (small subunit ribosomal RNA)	17
5S_rRNA	25
5_8S_rRNA	17

Table S5. Summary statistics of perfect microsatellites (SSR) predicted from *P. latus* genome Krait v1.4.0

Parameters	Description	Number
Total number of perfect SSRs	Counts	23,116
Total length of perfect SSRs	Bp	311,670
The average length of SSRs	Total SSR length/total SSR counts (bp)	13.49
SSRs per sequence	Total SSR counts/sequence counts	11,558
Percentage of sequence covered by SSRs	Total SSR length/total sequence length (%)	0.63
Relative abundance	Total SSRs/total valid length (loci/Mb)	470.45
Relative density	Total SSR length/total valid length (bp/Mb)	6,343.06

Table S6. Summary statistics of perfect microsatellite types predicted from the genome of *P. latus* Krait v1.4.0

Type	Counts	Length (bp)	Percent (%)	Average length (bp)	Relative abundance (loci/Mb)	Relative density (bp/Mb)
Di	73	1,106	0.32	15.15	1.49	22.51
Tri	324	5,541	1.4	17.1	6.59	112.77
Tetra	122	2,040	0.53	16.72	2.48	41.52
Penta	38	795	0.16	20.92	0.77	16.18
Hexa	26	3,090	0.11	118.85	0.53	62.89

Table S7. Comparison of the BUSCO assessment statistics of the broad mite, *P. latus* with previously sequenced mites, highlighting the low BUSCO completeness across the species

**BUSCO**	*P. latus*	*H. destructor*	*A. lycopersici*	*D. farinae*	*T. truncatus*	*T. urticae*
**Eukaryotic lineage**						
Complete BUSCOs (C)	92.9%	94.9%	86.3%	85.5%	94.1%	94.5%
Complete and single-copy BUSCOs (S)	92.5%	93.3%	85.1%	84.7%	91.4%	90.2%
Complete and duplicated BUSCOs (D)	0.4%	1.6%	1.2%	0.8%	2.7%	4.3%
Fragmented BUSCOs (F)	3.5%	2.0%	6.7%	7.8%	2.7%	2.4%
Missing BUSCOs (M)	3.6%	3.1%	7.0%	6.7%	3.2%	3.1%
Total BUSCO groups						255
**Arthropoda lineage**						
Complete BUSCOs (C)	84.3%	90.20%	69.60%	84.50%	90.20%	90.10%
Complete and single-copy BUSCOs (S)	83.4%	88.80%	68.00%	83.90%	85.90%	85.10%
Complete and duplicated BUSCOs (D)	0.9%	1.40%	1.60%	0.60%	4.30%	5.00%
Fragmented BUSCOs (F)	3.5%	2.60%	8.30%	6.40%	2.10%	2.10%
Missing BUSCOs (M)	12.2%	7.20%	22.10%	9.10%	7.70%	7.80%
Total BUSCO groups						1,013
**Arachnid lineage**						
Complete BUSCOs (C)	83.40%	89.40%	67.80%	91.70%	94.60%	94.60%
Complete and single-copy BUSCOs (S)	82.00%	87.80%	65.80%	90.40%	90.80%	89.80%
Complete and duplicated BUSCOs (D)	1.40%	1.60%	2.00%	1.30%	3.80%	4.80%
Fragmented BUSCOs (F)	1.60%	1.80%	1.50%	3.70%	0.70%	0.70%
Missing BUSCOs (M)	15.00%	8.80%	30.70%	4.60%	4.70%	4.70%
Total BUSCO groups						2,934
