# Supplementary material for: The miniature genome of broad mite, Polyphagotarsonemus latus (Tarsonemidae: Acari)
Source: Sci Data. 2024 Jul 9;11:748. doi: 10.1038/s41597-024-03579-4 (PMC11233664; doi:10.1038/s41597-024-03579-4)
Supplement: Supplementary file 2 — Fig. S1, Fig. S2, Fig. S3, Fig. S4, Fig. S5 [file 41597_2024_3579_MOESM2_ESM.pdf]

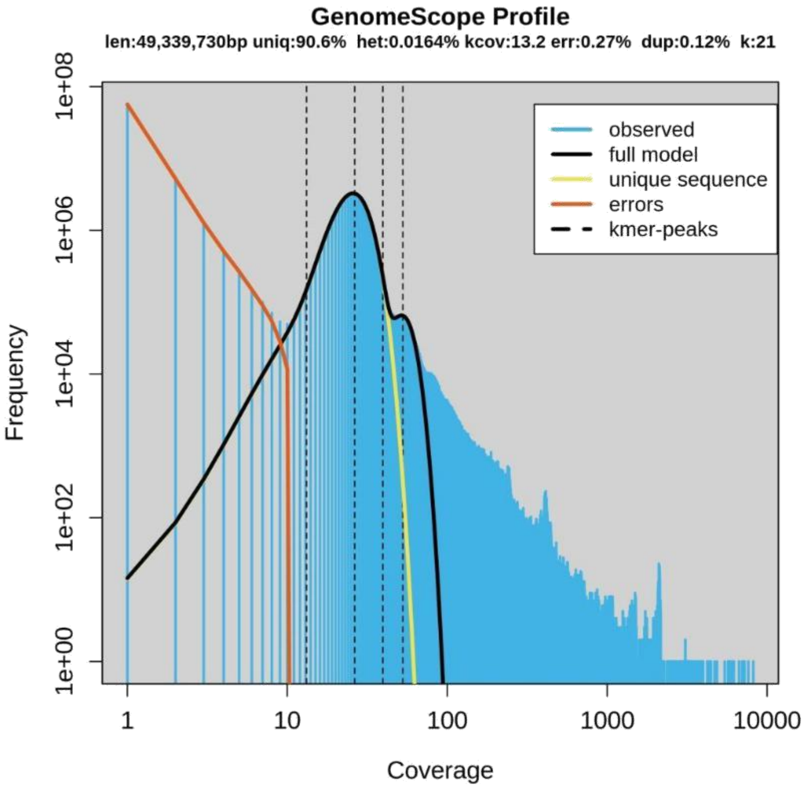

Fig. S1: k-mer frequency distribution plotted by GenomeScope from PacBio long reads for the estimation of genome size of *P. latus*. The distribution was determined with jellyfish using a k-mer size of 21. The X-axis represents the coverage of sequencing and the Y-axis indicates the frequency of coverage.

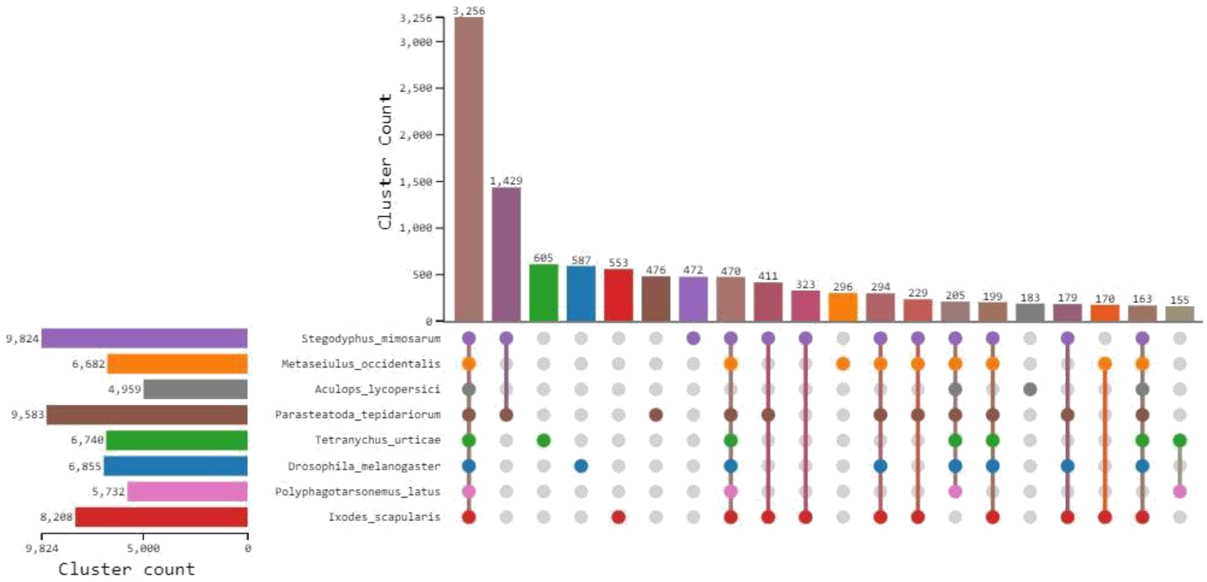

Fig. S2. Upset plot shows the distribution of shared and unique orthogroups between different arthropod species. A total of 14,646 clusters of orthogroups were identified. This plot visualizes the count of orthogroups associated with each species individually and those shared between species. The vertical bar graph depicts the number of orthogroups per species. The side plot provides an overview of the total number of orthogroups shared among species. The line represents the intersection. The species includes, African social velvet spider, *Stegodyphus mimosarum*; predatory mite, *Metaseiulus occidentalis*; tomato russet mite, *Aculops lycopersici*; common house spider, *Parasteatoda tepidariorum*; red spider mite, *Tetranychus urticae*; fruit fly, *Drosophila melanogaster*; broad mite, *Polyphagotarsonemus latus* and black legged tick, *Ixodes scapularis*

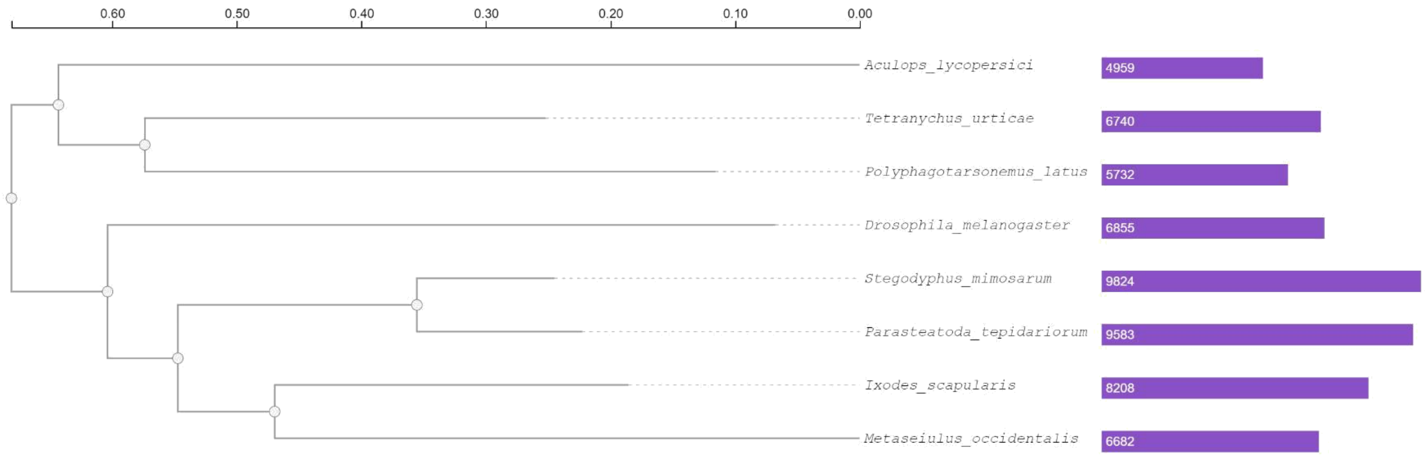

Fig. S3. Maximum likelihood phylogenetic tree of arthropod species which includes mite, spider and tick species constructed from 669 single copy clusters with dated nodes from time tree based on amino acid sequences shared by all focal taxa. The scale denotes the probability of distance.

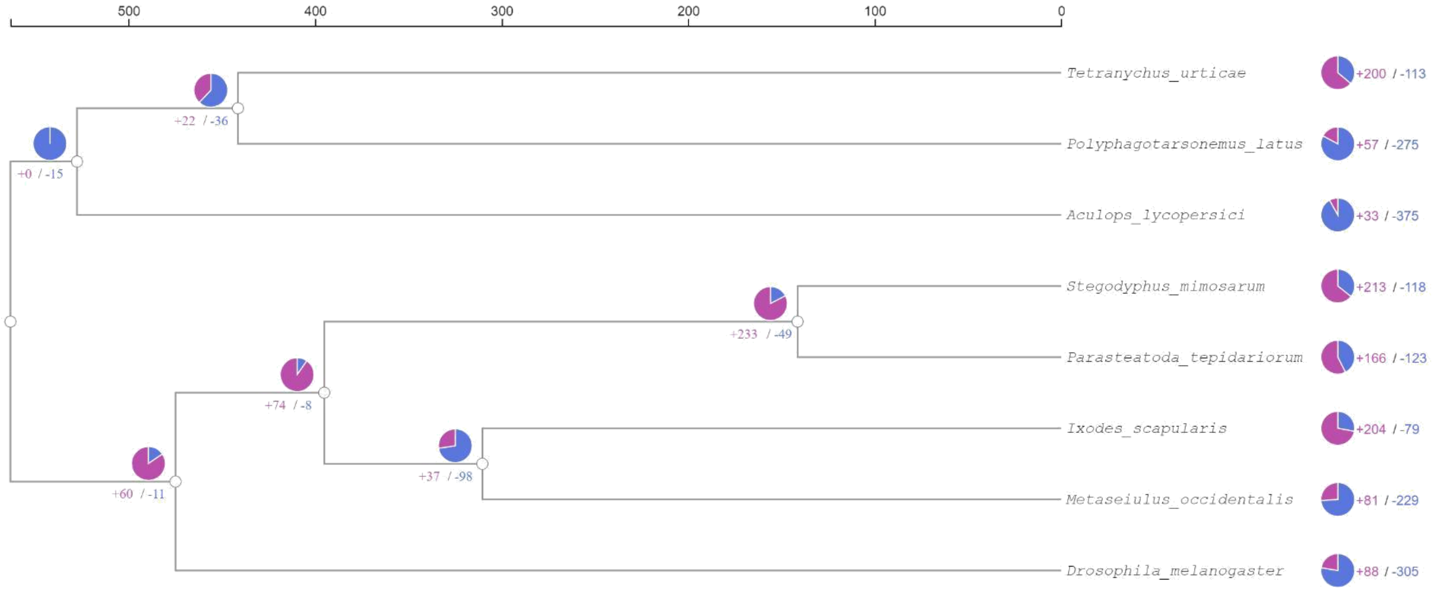

Fig. S4. Computational analysis of gene family evolution (CAFE) in Acari species estimated with *Drosophila melanogaster* as the out group. The loss and gain in number of genes are mentioned at the tip of nodes. Violet and blue colour denotes the expansion and contraction of gene families, respectively. All nodes have 100% bootstrap support and the scale denotes years in million.

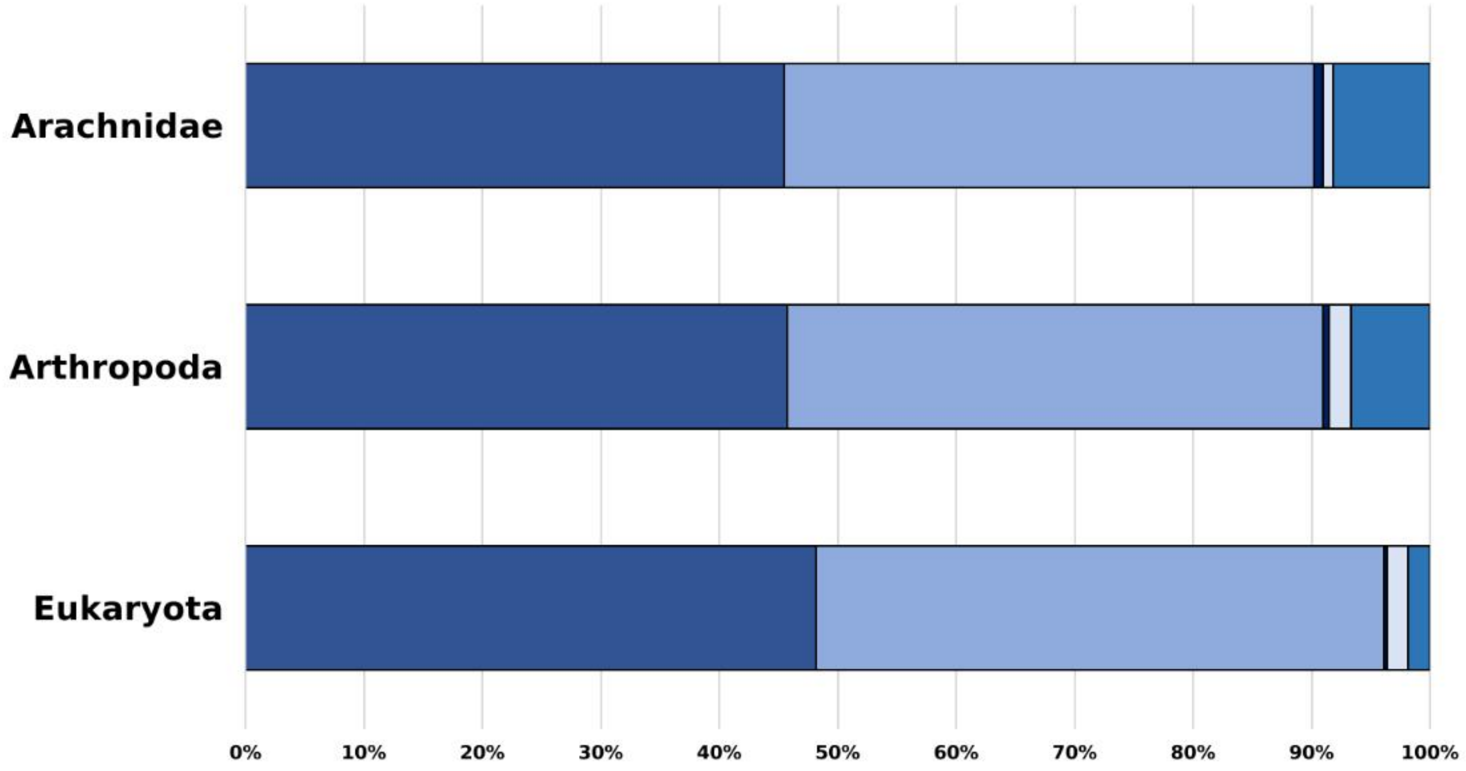

	Eukaryota	Arthropoda	Arachnidae
Complete BUSCOs(C)	237	854	2447
Complete and single-copy BUSCOs(S)	236	845	2406
Complete and Duplicated BUSCOs(D)	1	9	41
Fragmented BUSCOs(F)	9	35	47
Missing BUSCOs(M)	9	124	440

Fig. S5. BUSCO of *P. latus* using different BUSCO datasets (Eukaryota, Arthropoda and Arachnidae).
